# Effect of Hygrothermal Aging on the Mechanical Properties of Fluorinated and Nonfluorinated Clay-Epoxy Nanocomposites

**DOI:** 10.1155/2014/489453

**Published:** 2014-10-28

**Authors:** Salah U. Hamim, Raman P. Singh

**Affiliations:** School of Mechanical and Aerospace Engineering, College of Engineering, Architecture, and Technology, Oklahoma State University, Stillwater, OK 74078, USA

## Abstract

Hydrophilic nature of epoxy polymers can lead to both reversible and irreversible/permanent changes in epoxy upon moisture absorption. The permanent changes leading to the degradation of mechanical properties due to combined effect of moisture and elevated temperature on EPON 862, Nanomer I.28E, and Somasif MAE clay-epoxy nanocomposites are investigated in this study. The extent of permanent degradation on fracture and flexural properties due to the hygrothermal aging is determined by drying the epoxy and their clay-epoxy nanocomposites after moisture absorption. Significant permanent damage is observed for fracture toughness and flexural modulus, while the extent of permanent damage is less significant for flexural strength. It is also observed that permanent degradation in Somasif MAE clay-epoxy nanocomposites is higher compared to Nanomer I.28E clay-epoxy nanocomposites. Fourier transform infrared (FTIR) spectroscopy revealed that both clays retained their original chemical structure after the absorption-desorption cycle without undergoing significant changes. Scanning electron microscopy (SEM) images of the fracture surfaces provide evidence that Somasif MAE clay particles offered very little resistance to crack propagation in case of redried specimens when compared to Nanomer I.28E counterpart. The reason for the observed higher extent of permanent degradation in Somasif MAE clay-epoxy system has been attributed to the weakening of the filler-matrix interface.

## 1. Introduction

Epoxy polymers are very important class of advanced materials. Their main distinction from other types of polymers lies in their densely crosslinked molecular structure. This crosslinking leads to a number of favorable thermal and mechanical properties including high strength and modulus, high creep resistance, high glass transition temperature, low shrinkage, and better chemical resistance. These properties in conjunction with ease of processing have made epoxy resins an attractive choice for use in many engineering components and structures. They have found huge applications in aerospace, automotive, packaging, coating, and microelectric industries. In recent years, researchers have developed and investigated polymer nanocomposites based on a wide variety of micro-/nanoscale fillers including clay particles [1–6], aluminum particles [7], TiO_2_ particles [8], graphenes [9, 10], carbon nanotubes [11–13], and halloysites [4, 14] to improve the mechanical properties of epoxy polymers. Among the various reinforcements, large aspect ratio layered silicates or clays are especially attractive for enhancing the barrier properties and, hence, can be used to improve the resistance to moisture degradation.

Epoxy polymers are characteristically hydrophilic, which means that they have strong affinity towards water. This nature of epoxy resins makes them susceptible to high moisture absorption; in general, depending on the nature of the epoxy resin, the equilibrium moisture uptake can be in the range of 1–7% [15]. Absorbed moisture usually degrades the functional, structural, and mechanical properties of the polymer matrix [4, 16–19]. It has been reported that mechanical and thermal properties of epoxy-based systems are severely affected by moisture absorption in comparison to other matrix materials, such as bismaleimide (BMI), polyetheretherketone (PEEK), and cyanate ester [20].

Water absorption into a polymer matrix leads to change in both chemical and physical characteristics and affects the mechanical properties through different mechanisms such as plasticization, crazing, hydrolysis, and swelling. Plasticization is the most important physical change that occurs through the interaction of the water molecules with polar groups in the matrix, which can severely depress the glass transition temperature [12, 18, 21–24]. For example, high moisture absorption capability of TGDDM/DDS epoxy resin, which is about 7 wt%, reduces the system T_g_ from 260°C to 130°C [25–27]. In general, for most epoxy systems, T_g_ is reduced by 20°C/1 wt% of moisture intake [28]. The decrease in modulus of epoxy has also been reported to be due to plasticization according to several studies [29–32]. Other studies showed that the decrease in modulus resulted from crazing [33–35], where the absorbed water acted as a crazing agent continuously decreasing the mechanical strength of epoxies with exposure time in water [34]. This was supported by SEM micrographs of epoxies, which have shown cavities and fractured fibrils that could be explained by a moisture induced crazing mechanism [33]. The aforementioned chemical changes mainly include chain scission and hydrolysis [22, 36]. Plasticization is considered reversible upon drying, while other effects of moisture absorption are irreversible.

In recent years, effect of moisture absorption on the mechanical properties of neat epoxy and clay-epoxy nanocomposite has been frequently studied. Zhao and Li reported that tensile strength and modulus decreased for both neat epoxy and nanocomposites upon moisture absorption, while the tensile strain increased significantly for moisture absorbed samples. Although failure occurred in brittle manner, effect of plasticization was found in SEM images, which showed shear yielding for both neat epoxy and nanocomposite samples after being exposed to moisture [37]. Similar degradation in mechanical properties has been reported by Glaskova and Aniskevich [38]. Alamri and Low reported lower flexural strength and modulus for halloysite, nanoclay, and nanosilicon carbide nanocomposites as a result of moisture absorption [4]. Al-Qadhi et al. studied the effect of moisture absorption on the tensile properties of clay-epoxy nanocomposites and found that tensile strength and modulus decrease as a result of water uptake [24]. Wang et al. investigated the effect of hydrothermal degradation on mechanical properties such as tensile strength, modulus, and fracture toughness with immersion duration [39]. For DGEBA epoxy systems, fracture toughness and modulus were not influenced much with immersion time, while tensile strength decreased for nanocomposites. Tensile and flexural properties of nanoclay reinforced composite laminates and CNT reinforced nanocomposites have also been found to be affected adversely as a result of moisture absorption [12, 40]. According to the study conducted by Buck et al., at elevated temperature, combination of moisture and sustained load significantly reduced ultimate tensile strength of E-glass/vinyl-ester composite materials [41]. A study on elastic modulus of epoxy polymer after absorption-desorption cycle showed recovery of property from wet condition, although modulus remained at a lower value than the as-prepared samples for lower filler volume. For higher volume fraction of reinforcement, elastic modulus improved to a value which was more than the elastic modulus of the as-prepared samples [19]. Phua et al. also reported recovery of tensile properties after refrying OMTT-PBS nanocomposites. A similar study conducted by Ferguson and Qu showed recovery of elastic properties from moisture saturated state after a desorption cycle [42]. However, de'N$\overset{`}{\text{e}}$ve and Shanahan did not observe any recovery of elastic modulus after absorption-desorption cycle at elevated temperature [22]. It is evident from the published works that moisture absorption can severely alter mechanical properties of epoxies by decreasing the elastic modulus [12, 29, 33], tensile strength [3, 12], shear modulus [30, 31], flexural strength and flexural modulus [40, 43], yield stress, and ultimate stress [32] as water uptake increases.

Most of the research on polymer-clay nanocomposites has been mainly focused on investigating the effect of moisture absorption on mechanical properties such as elastic modulus and tensile strength. Although fracture toughness is a very important property for these nanocomposites as these are used in various structural applications, the effects of moisture absorption on fracture toughness of polymer-clay nanocomposites have not been studied extensively. Durability of polymer/clay nanocomposites is still needed to be studied in depth, particularly for hygrothermal aging in which the degradation of the mechanical properties and loss of integrity of these nanocomposites occur from the simultaneous action of moisture and temperature. This study on clay-epoxy nanocomposites was designed to investigate the effect of hygrothermal aging on mechanical properties of these nanocomposites. Two different clay particles were used to investigate the effect of clay structure on the permanent property changes due to hygrothermal aging. A drying cycle was employed to quantify the recovery of the properties after hygrothermal aging. This was helpful to understand the extent of permanent degradation that occurred by the combined application of elevated temperature and moisture. Mechanical properties in terms of fracture toughness, flexural strength, and flexural modulus are the properties that were studied. Scanning electron microscopy and Fourier transform spectroscopy were conducted to further elucidate the underlying fracture mechanisms of these preconditioned specimens.

## 2. Materials and Characterization

The epoxy resin used for this study is EPON 862, which is a diglycidyl ether of bisphenol F. The curing agent used for this resin system was a moderately reactive, low viscosity aliphatic amine curing agent, Epikure 3274. Both of these chemicals were supplied by Miller-Stephenson Chemical Company, Inc., Dunbury, Connecticut. Two structurally different clay particles were used as reinforcement. Nanomer I.28E is a surface modified montmorillonite based layered silicate (Nanocor, Inc., Arlington Heights, IL) modified with a quaternary amine (trimethyl stearyl). Somasif MAE (Co-Op Chemical Co., Japan), which was the other clay particle used for this study, is a synthetic mica modified with dimethyl dihydrogenated tallow ammonium chloride.  Table 1 shows the structural composition of the clay particles used in this study.

### 2.1. Sample Preparation

Epoxy was preheated to 65°C before desired amount of clay was introduced and mixed using mechanical mixer for 12 hours. To reduce the viscosity of the mixture and to facilitate mixing, temperature was maintained at 65°C using a hot plate for the entire duration of mixing. The mixture was then degassed for around 30 minutes to remove any entrapped air bubbles. Bubble-free mixture of clay and epoxy was then shear-mixed using a high-speed shear disperser (T-25 ULTRA TURRAX, IKA Works Inc., North Carolina, USA) for 30 minutes. During this process, temperature was maintained at 65°C using an ice bath. Subsequently, the mixture was then degassed until it was completely bubble-free. Curing agent was added to the mixture at 100 : 40 weight ratio and carefully hand-mixed to avoid introduction of any air bubble. After it was properly mixed, the final slurry containing epoxy and clay was poured into an aluminum mold and cured at room temperature for 24 hours followed by postcuring at 121°C for 6 hours. The final sample had a nominal dimension of 177.80 mm × 152.50 mm × 12.70/6.35 mm. To study the effect of loading percentage, the weight fraction of the clay in the nanocomposite was varied from 0.5 to 2.0%.

### 2.2. Environmental Preconditioning

After specimens were cut into the final required dimension according to the ASTM standards D5045 and D790, they were subjected to degradation. Specimens from each nanocomposite were taken and submerged in purified deionized boiling water for 24 hours. Water saturated specimens were dried in an oven at 110°C for 6 hours to remove moisture from the samples leaving only permanent degradation in the form of bonded water.

### 2.3. Fracture Toughness,* K*_Ic_, Determination

Critical stress intensity factor, *K*_Ic_, was determined by single edge notch bend (SENB) test as per the ASTM D5045 on universal testing machine (Instron 5567, Norwood, MA) in a displacement-controlled mode with fixed crosshead speed of 10 mm/min. Nominal dimension for the SENB test samples was 67.30 mm × 15.20 mm × 6.35 mm. A notch was created using precision diamond saw (MK-370, MK Diamond Products Inc., Torrance, CA, USA) and, afterwards, a sharp precrack with ratio of 0.45 < *a*/*W* < 0.55 was created by tapping a fresh razor blade into the notch. At least 5 specimens were tested for every condition. Fracture toughness for the specimens was calculated in terms of critical stress intensity factor, *K*_Ic_. The crack length, *a*, was measured using an optical microscope (Nikon L150) which has a traveling plate with graduations:

(1) $$\begin{array}{c} K_{\text{Ic}}=\frac{P}{B\sqrt{W}}f\left(\frac{a}{W}\right), \end{array}$$

where *P* = maximum applied force (N), *B* = thickness of the specimen (mm), *W* = width of the specimen (mm), and *f*(*a*/*W*) = geometry factor, and it is given by the following equation:

(2) f(aW)=3(S/W)a/W2(1+2(a/W))(1−a/W)3/2 ×[(2.15−3.93(aW)+2.7(aW)2)1.99−(aW)(1−aW)iiiiiii×(2.15−3.93(aW)+2.7(aW)2)],

where *S* = support span (mm) and *a* = length of the precrack (mm).

### 2.4. Flexural Strength and Flexural Modulus Determination

Flexural strength and flexural modulus were determined using three-point bend (3PB) test according to ASTM D790 on universal testing machine (Instron 5567, Norwood, MA). The nominal dimension for the flexural test specimens was 55.90 mm × 12.70 mm × 6.35 mm. The crosshead speed for the test was calculated using (3). The crosshead speed was found to be 1.35 mm/min. Consider

(3) $$\begin{array}{c} R=\frac{ZL^{2}}{6d}, \end{array}$$

where *R* = rate of crosshead motion (mm/min), *L* = support span (mm), *d* = depth of beam (mm), and  *Z* = rate of straining of the outer fiber (mm/mm/min) = 0.01.

The flexural strength and flexural modulus were calculated using the following equations, respectively:

(4) $$\begin{array}{c} \sigma_{f,\max}=\frac{3P_{\max}L}{2bd^{2}}, \end{array}$$

(5) $$\begin{array}{c} E_{b}=\frac{L^{3}m}{4bd^{3}}, \end{array}$$

where *σ*_*f*,max⁡_ = flexural strength (MPa), *E*_*b*_ = flexural modulus (MPa), *P*_max⁡_ = maximum load on the load-deflection curve (N), *L* = support span (mm), *b* = width of beam tested (mm), *d* = depth of beam tested (mm), and *m* = slope of the tangent to the initial straight-line portion of the load-deflection curve (N/mm of deflection).

### 2.5. Fracture Surface Morphology

Surface morphology of the fractured specimens from SENB tests was observed using scanning electron microscopy (Hitachi S-4800 FESEM, Dallas, TX). As polymer materials are nonconductive to electrons, all fracture surfaces were sputtered with gold-palladium alloy before SEM imaging.

### 2.6. Fourier Transform Infrared Spectroscopy

FTIR spectroscopy measurements were performed using ATR-FTIR spectrometer (Nicolet iS10, Waltham, MA) using 64 scans at a resolution of 2.0 cm^−1^. Each spectrum was recorded from 4000 to 500 cm^−1^ at room temperature. Spectra were analyzed using OriginPro 9.0 (OriginLab, Northampton, MA).

## 3. Results and Discussions

### 3.1. Gravimetric Measurements

Table 2 shows the relative weight changes that occurred in the specimens after 24 hours of boiling water absorption and 6 hours of high temperature desorption cycle. For the studied material systems, percentage weight gain after absorption cycle showed no change as a function of clay loading percentage. This observation was different from the findings reported by Glaskova and Aniskevich for clay-epoxy nanocomposites [44]. According to their study, moisture absorption was found to have increased slightly with the increase of clay weight percentage. Contrarily, Alamri and Low reported decreasing moisture absorption with increasing clay weight percentage [4]. The reason why a different clay-epoxy nanocomposite system behaves differently in moisture absorption test is still not clear and further investigation is required to understand it. In this study, although both clays are structurally different, the observed percentage weight gain for both nanocomposite systems was found approximately to be the same. These two observations led to the conclusion that moisture diffusion process primarily depended on the polymer system under investigation. Moisture desorption data showed that most of the absorbed water is free water, which can be driven out of the system by drying. For neat epoxy, the amount of retained water after desorption cycle is less compared to the nanocomposites. This is possibly due to the fact that presence of clay hindered the moisture diffusion process in and out of the epoxy polymers. An increasing trend in the amount of retained moisture for higher clay loading nanocomposites also supported the aforementioned statement. Amount of water retained after the desorption cycle has been found to be almost similar for both clay-epoxy nanocomposite systems.

### 3.2. Fracture Toughness

**Figure 1:**
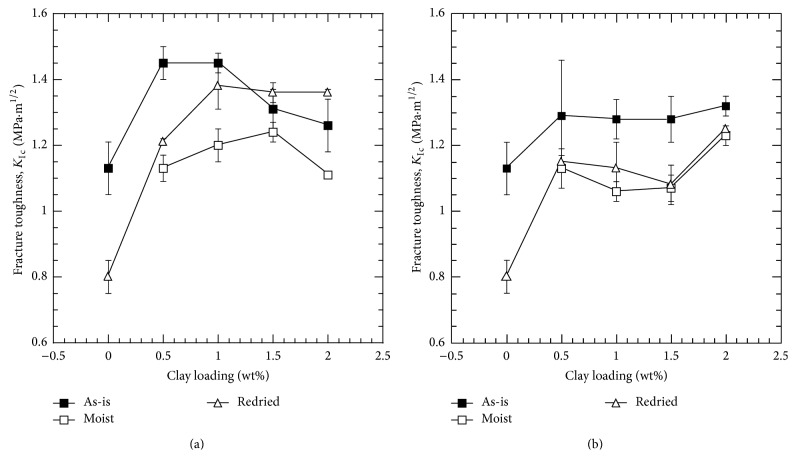
Critical stress intensity factor, *K*_Ic_, as a function of clay loading: (a) Nanomer I.28E and (b) Somasif MAE clay-epoxy nanocomposites.

The critical stress intensity factors as a function of clay loading percentage for Nanomer I.28E and Somasif MAE clay-epoxy nanocomposites are shown in Figure 1. *K*_Ic_ values for the as-prepared samples are also listed as a reference.

Critical stress intensity factor, *K*_Ic_, increased 28% for the as-prepared 0.5 wt% Nanomer I.28E clay-epoxy nanocomposite compared to neat epoxy. The reason behind this observation can be attributed to the layered structure of the clay. Clay in a polymer material physically blocks, bifurcates, and deflects the crack path, compelling the crack to travel longer path, which in turn results in higher toughness in a clay-polymer nanocomposite. The toughening effect of clay on epoxy polymer started to decrease with any additional clay reinforcement. This is a common behavior for several types of epoxy-clay nanocomposites and has been reported in previous studies conducted on clay-epoxy nanocomposites [3, 6, 45, 46]. Depending on the processing technique and epoxy-clay interaction, there is an optimum weight percentage for which the property enhancement can be maximized. Any further addition of clay negates the positive effect by forming agglomerates due to improper exfoliation of the clay platelets and thus results in stress concentration forcing the material to fail at lower loads. For moisture saturated epoxy-clay nanocomposites, *K*_Ic_ was found to be lower compared to the as-prepared nanocomposite samples for all the Nanomer I.28E nanocomposites. As water molecules diffuse into the nanofiller-epoxy interface, debonding and weakening of the interface occur, resulting in poor stress transfer between the filler and the epoxy matrix [43, 47, 48]. Redried neat EPON 862, free of void-filling water, showed 29% reduction in fracture toughness compared to the as-prepared neat EPON 862 samples. Addition of 0.5 wt% of clay resulted in 16% reduction in fracture toughness when compared to the as-prepared samples, which was significantly less compared to 29% reduction of neat epoxy. For 2.0 wt% Nanomer I.28E clay-epoxy nanocomposite, redried samples were found to be tougher than the as-prepared samples. However, the standard deviation of the as-prepared sample was much higher, which could possibly mean that the dried and the as-prepared samples have no difference in toughness.

For the as-prepared and moisture saturated samples, Somasif MAE nanocomposites showed comparable trend in fracture toughness data; clay reinforcement successfully improved the baseline epoxy properties and moisture absorption degraded the mechanical properties for all clay percentages. However, the permanent degradation after absorption-desorption cycle was found to be more prominent in the case of Somasif MAE clay nanocomposites compared to Nanomer I.28E clay nanocomposites. The recovery of property after 6 hours of drying was negligible for Somasif MAE clay nanocomposites, whereas Nanomer I.28E nanocomposites showed significant recovery of property after drying. The difference in property recovery of these two clay-epoxy nanocomposites can be attributed to the structural differences of the two clay particles and has been further investigated through SEM and FTIR technique.

### 3.3. Flexural Properties

**Figure 2:**
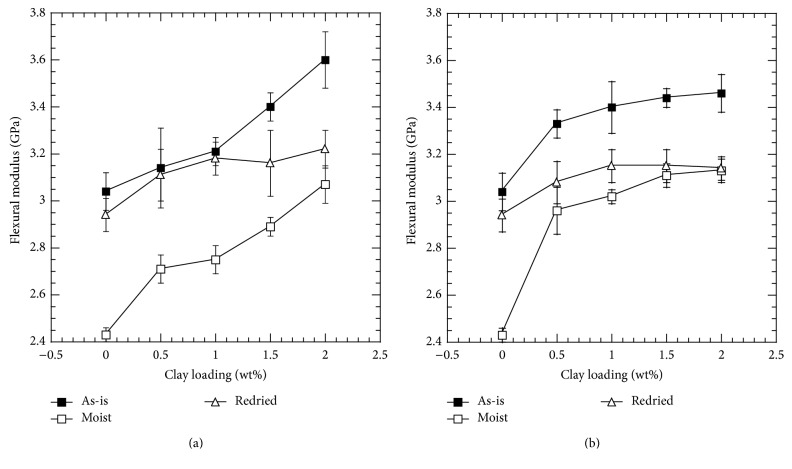
Flexural modulus as a function of clay loading: (a) Nanomer I.28E and (b) Somasif MAE clay-epoxy nanocomposites.

Flexural modulus for the neat epoxy and clay-epoxy nanocomposites was determined from 3PB test and has been plotted against clay loading percentage in Figure 2 for Nanomer I.28E and Somasif MAE clay. Flexural modulus increased almost linearly for both clays as a function of clay loading percentage. According to previous studies on epoxy polymers, incorporation of hard substance such as clay in polymer matrix results in higher modulus [49]. When a load is applied on epoxy, the polymer chains slide past each other and deform. This deformation is higher in less crosslinked structures compared to higher crosslinked structures. Once layered silicates such as clay particles are introduced in a polymer system, it restricts the motion of the polymer chain sliding and makes the matrix less pliable. As the clay content increases, it is more difficult for the polymer chains to untangle and move. This increase in restriction of polymer chains is responsible for the increase in modulus as the clay percentage increases.

For the hygrothermally conditioned specimens, the modulus is lower compared to the as-prepared specimens. This behavior observed is mostly due to the presence of water inside the epoxy system, which increases the ductility of the epoxy system. Water acts as an effective plasticizer and can diffuse into the nanofiller-polymer interface and weaken the bonding between them [40, 43]. Presence of water in epoxy system also results in an increase in free volume through rupture of hydrogen bonding between polymer chains, which increases the chain mobility and eases the segmental motion when a load is applied to the composite [50]. These physical changes can be attributed to the observed lower modulus for water absorbed specimens. Other mechanisms affecting the polymer such as hydrolysis and chain scission may also be responsible for lowering the modulus. Once hydrolysis and chain scission take place, less bonding between the polymers makes it more deformable resulting in lower modulus for aged samples [22, 36, 51]. The effect of hygrothermal aging was more severe in neat epoxy than in the nanocomposites. For neat EPON 862, flexural modulus decreased 20% after hygrothermal aging, whereas it was only 13% and 11% for 0.5 wt% of Nanomer I.28E and Somasif MAE clay-epoxy nanocomposites, respectively. In addition to being hard substance, clay particles have very high aspect ratio. The high aspect ratio of the clay platelets provides resistance against polymer chain mobility in a water absorbed ductile polymer leading to the observed lower degradation of flexural modulus values in comparison to neat polymer.

For Nanomer I.28E clay-epoxy samples conditioned at 110°C for 6 h, recovery of flexural modulus was observed. Once redried, free water residing in the microvoids was evaporated, and the effect of plasticization was not prominent anymore. As a result, the ductility of the polymer reduced and the recovery of mechanical properties from moisture absorbed state occurred. Nevertheless, in case of Somasif MAE clay-epoxy samples, modulus recovery was negligible after the desorption cycle. Due to the structural difference between the two clay particles, it is possible that the interface of Somasif MAE clay-epoxy is being more affected by the hygrothermal degradation than the Nanomer I.28E clay-epoxy interface.

**Figure 3:**
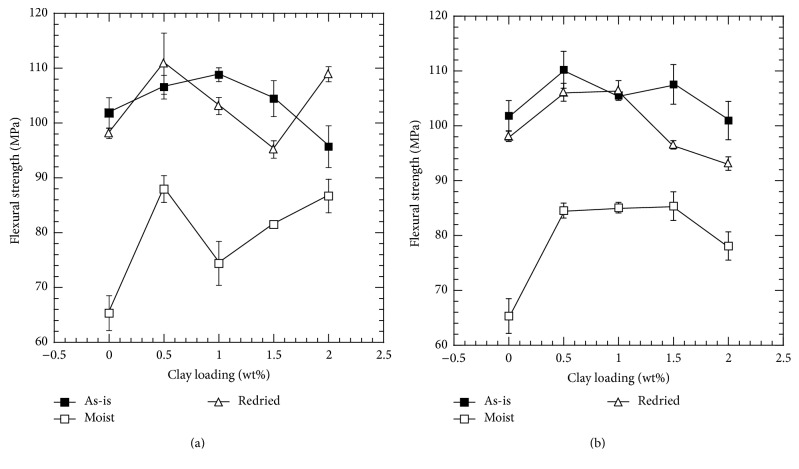
Flexural strength as a function of clay loading: (a) Nanomer I.28E and (b) Somasif MAE clay-epoxy nanocomposites.

Flexural strengths for the epoxy and clay-epoxy nanocomposites were determined using three-point bend (3PB) test and are plotted against clay loading percentage in Figure 3 for Nanomer I.28E and Somasif MAE clay. Figure 3 shows that the addition of Nanomer I.28E clay provided negligible improvement in flexural strength compared to neat epoxy. The maximum improvement in flexural strength was found to be less than 10% for both clay-epoxy systems from the base flexural strength of neat epoxy. Similar observation has been reported in the literature, where addition of nanoclay particles did not significantly improve the flexural strength of the system [52]. Furthermore, when 2.0 wt% of clay is added to the nanocomposite, flexural strength value dropped to a lower value than the neat epoxy. Increasing the amount of nanoparticles more than a certain amount has been found to reduce the flexural strength in earlier studies [53]. It can also be observed that moisture absorbed nanocomposites showed significant reduction in flexural strength. For 1.0 wt% of I.28E clay-epoxy nanocomposites, reduction in flexural strength due to hygrothermal aging is 32%. Reduction in flexural strength of nanocomposites after moisture absorption has been previously reported in the literature [4, 43, 54–56] and has been attributed to the degradation of interface region, which in turn reduces stress transfer between the nanofiller and the matrix. For redried samples, as most of the moisture is driven out of the system and plasticization effect was minimal, flexural strength for these samples recovered almost fully. For instance, flexural strength recovers to 95% of its original value for 1.0 wt% of Nanomer I.28E clay-epoxy nanocomposite.

Almost similar trend was observed for Somasif MAE clay-epoxy nanocomposites, where addition of clay did not change the property significantly, and after 24 h of hygrothermal aging property decreased to a lower value compared to the as-prepared samples. However, the severity of degradation was much less in both clay-epoxy systems compared to neat epoxy system. Well dispersed high aspect ratio clay platelets have the capability of crack deflection and crack arresting, which can lead to the observed higher flexural strength in wet clay-epoxy samples in comparison to neat epoxy samples [48, 57]. For redried nanocomposites, similar trend was observed for both material systems and it was found that flexural strength recovers almost fully. In this study, flexural strength has not been largely affected by the addition of clay into the epoxy. This may as well mean that flexural strength has been primarily governed by the flexural strength of the epoxy. As the strength of neat epoxy was marginally affected by the absorption-desorption cycle, so did the strength of clay-epoxy nanocomposites.

### 3.4. Scanning Electron Microscopy (SEM)

**Figure 4:**
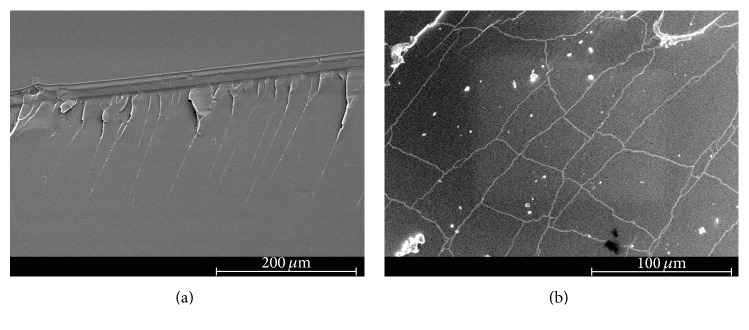
Scanning electron micrographs of neat epoxy polymer: (a) as-prepared and (b) redried.

**Figure 5 fig5:**
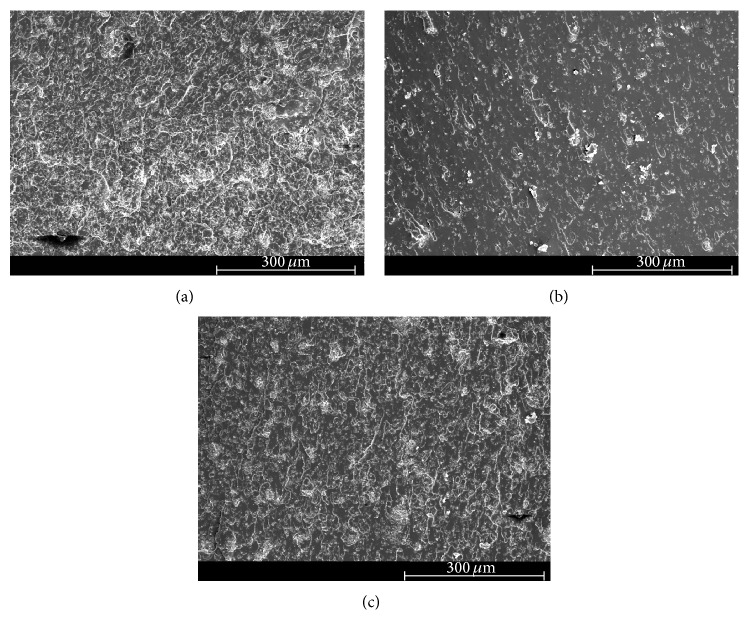
Scanning electron micrographs of the crack growth region for 0.5 wt% Nanomer I.28E clay-epoxy nanocomposites: (a) as-prepared, (b) moisture absorbed, and (c) redried.

**Figure 6 fig6:**
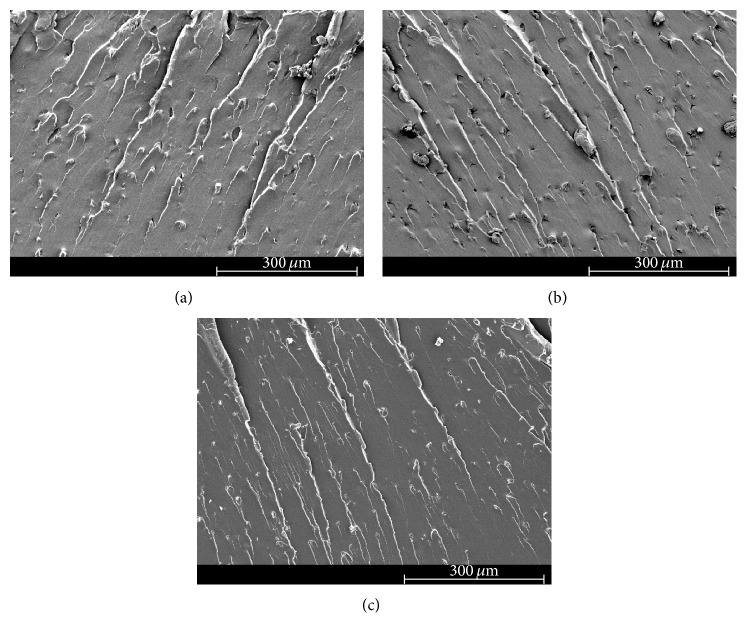
Scanning electron micrographs of the crack growth region for 0.5 wt% Somasif MAE clay-epoxy nanocomposites: (a) as-prepared, (b) moisture absorbed, and (c) redried.

**Figure 7 fig7:**
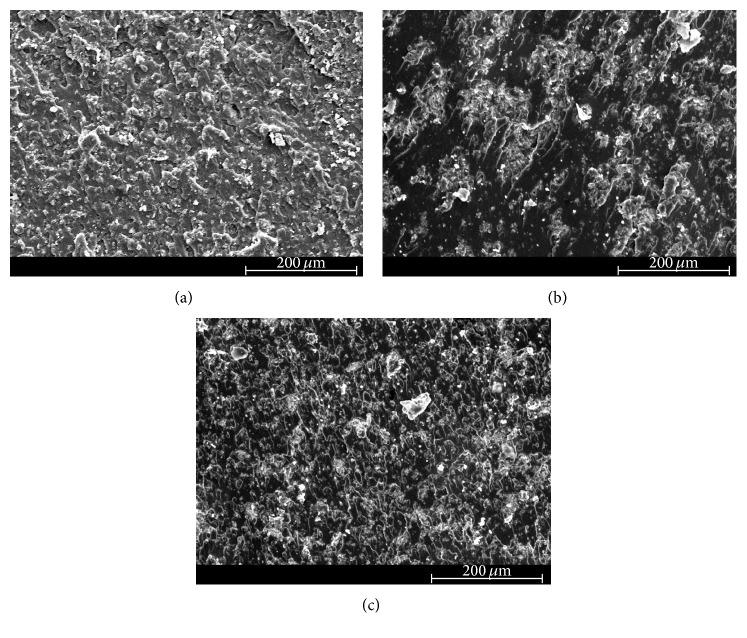
Scanning electron micrographs of the crack growth region for 1.5 wt% Nanomer I.28E clay-epoxy nanocomposites: (a) as-prepared, (b) moisture absorbed, and (c) redried.

**Figure 8:**
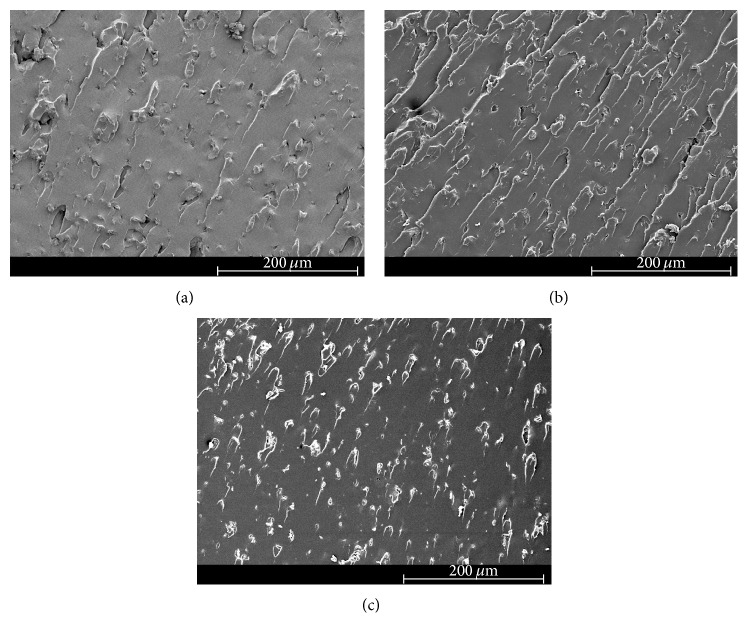
Scanning electron micrographs of the crack growth region for 1.5 wt% Somasif MAE clay-epoxy nanocomposites: (a) as-prepared, (b) moisture absorbed, and (c) redried.

The scanning electron micrographs of the fracture surface of tested neat epoxy and clay-epoxy nanocomposites are shown in Figures 4 [Fig fig5] [Fig fig6] [Fig fig7]–8. SEM micrograph of the fracture surface of the as-prepared neat polymer (Figure 4(a)) showed characteristic brittle failure with a smooth fracture surface. This mirror-like fracture surface is an indication of poor fracture toughness of epoxy and has been reported in previous studies conducted on epoxy polymers [6]. For the redried neat epoxy specimen (Figure 4(b)), a network of microcracks throughout the fracture surface is found. For TGDDM-DDS system, Morgan et al. observed similar behavior [58]. According to their study, absorbed moisture enhances craze initiation and propagation in polymer which can result in the formation of microcracks or fibrils in the polymer system. In this study, lower fracture toughness for dried neat polymer compared to unaged neat polymer can be attributed to the formation of these microcracks.

The SEM micrographs of clay incorporated epoxy systems showed significantly rougher fracture surface compared to the neat polymer. Clay, if present in a system, physically blocks and slows down the crack propagation and the resulting fracture surface of the nanocomposite shows river-markings instead of smooth fracture surface found in the neat polymer. These river-markings provide clear indication of the enhanced toughening mechanism in polymeric materials due to clay incorporation and support the observed higher fracture toughness for clay-epoxy nanocomposites compared to the neat epoxy polymer. Fracture surfaces of nanofiller reinforced polymer nanocomposites showing higher surface roughness have been reported in prior studies [4, 6, 59]. Comparing the fracture surfaces of the as-prepared Nanomer I.28E and Somasif MAE nanocomposite systems (Figures 5(a), 6(a), 7(a), and 8(a)), it was observed that the as-prepared Somasif MAE clay nanocomposite has considerably less amount of river-markings, which is indicative of poor toughening in Somasif MAE clay nanocomposites. This observation supported the difference of critical stress intensity factor measured for these two nanocomposite systems. Poor adhesion between Somasif MAE clay and epoxy resulted in less energy requirement during new surface formation, which can be attributed as the reason of these nanocomposites showing less fracture toughness than Nanomer I.28E clay nanocomposites for the same clay loading percentage.

Fracture surface of moisture absorbed Nanomer I.28E clay nanocomposites (Figures 5(b) and 7(b)) showed less number of river-markings (i.e., lower critical stress intensity factor) than the as-prepared nanocomposites. SEM micrograph of Somasif clay-epoxy nanocomposites (Figures 6(b) and 8(b)) showed the presence of shear leaps. As shear yielding requires less energy to form new surface, moisture absorbed specimens had lower fracture toughness than the as-prepared specimens. Although shear yielding was found to be the principle mechanism of failure in these specimens, some form of crack bifurcation and crack pinning was also present in these fracture surfaces.

Fracture surface micrographs of redried Nanomer I.28E nanocomposites (Figures 5(c) and 7(c)) showed more roughness than the moisture absorbed specimens indicating higher fracture energy absorbance for these specimens. Micrographs of redried Somasif MAE clay-epoxy samples (Figures 6(c) and 8(c)) showed significantly less rough fracture surface than the redried Nanomer I.28E samples. It is important to note that these redried Somasif MAE clay-epoxy fracture surfaces showed very little resistance against the crack propagation even after most of the water was driven out of the system. This observation led to the speculation that absorbed moisture could have weakened the interface of the Somasif MAE clay particles and the epoxy matrix. The replacement of –OH (hydroxyl) groups from the octahedral layer of the clay by the –F (fluorine) groups makes the Somasif MAE clay particles much more hydrophobic compared to the Nanomer I.28E clay particles. The higher hydrophobicity of the Somasif MAE clay particles could have exerted an additional force on the moisture absorbed interface and weakened the bond between the clay particles and the epoxy chains. This might have in turn resulted in the poor adhesion/less fracture energy absorption in the redried Somasif MAE clay-epoxy nanocomposites.

### 3.5. Fourier Transform Infrared (FTIR) Spectroscopy 

**Figure 9:**
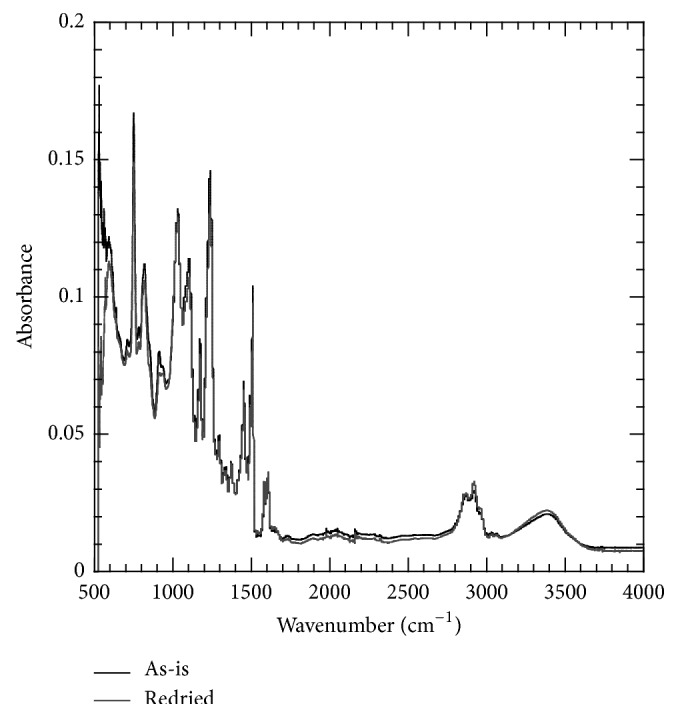
FTIR spectra for neat epoxy polymer before and after absorption-desorption cycle.

FTIR spectra of the as-prepared and redried neat epoxy are shown in Figure 9. From the FTIR spectra, it was evident that as a result of the absorption-desorption cycle the epoxy underwent some permanent chemical changes. These chemical changes were probably due to the hydrolysis and chain scission mechanism. The drying cycle used in this study removed most of the water that was absorbed by the polymer system, and it was believed that the remaining water (only about 7% of the total moisture uptake) is chemically bound to the polymer system. FTIR spectra (Figure 7) showed band at 3200–3400 cm^−1^, which is characteristic OH stretching of the hydroxyl group. However, significant difference was not observed for the bands at 3200–3400 cm^−1^.

**Figure 10:**
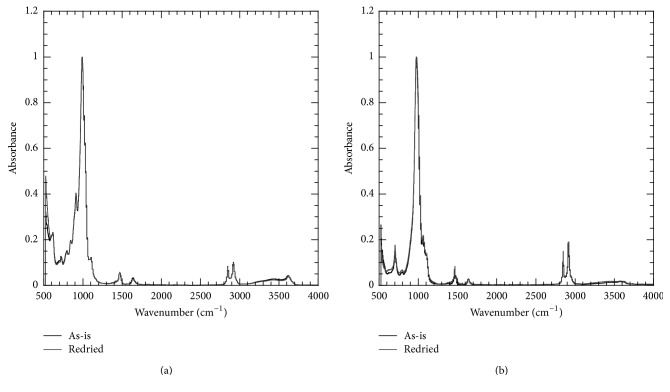
FTIR spectra for clay particles before and after absorption-desorption cycle: (a) Nanomer I.28E and (b) Somasif MAE.

Figure 10 shows the FTIR spectra for Nanomer I.28E and Somasif MAE clay powder before and after the absorption-desorption cycle. Both clays showed characteristic Si–O peak in the 990 cm^−1^ region. Nanomer I.28E clay showed another peak at 915 cm^−1^ region, which is characteristic Al–OH peak. In Somasif MAE, the OH groups present in the corner of the octahedral layer of Nanomer I.28E were substituted with F, which explains the absence of the peak at 915 cm^−1^. Both clays in as-is condition showed 3200–3400 cm^−1^ band for hydroxyl groups. However, the band was weaker in case of Somasif MAE compared to Nanomer I.28E clay because of the hydrophobic nature of the Somasif MAE clay. It was interesting to note that the overall changes the clay powders underwent as a result of absorption-desorption cycle are fairly small and can be considered as insignificant.

## 4. Conclusion

Property deterioration due to moisture absorption has been one of the most important areas of interest in polymer research for the last few years. To be used as structural materials, it is of utmost importance to understand the reversible and irreversible changes occurring in polymeric materials as a result of moisture absorption. This study was conducted to elucidate the effect of moisture absorption on the mechanical properties of clay reinforced epoxy polymers. Fracture toughness, flexural strength, and flexural modulus were determined for two different clay-epoxy nanocomposites following the ASTM standards. The effects of hygrothermal aging and subsequent redrying on the mechanical properties of these polymer nanocomposites were investigated. After removing the free water by drying, the irreversible effect or the permanent damage due to hygrothermal aging on the clay-epoxy nanocomposite systems was determined. Irrespective of the clay reinforcement type, all the studied properties were degraded due to hygrothermal aging. Several physical and chemical changes, such as interface weakening, hydrolysis, and chain scission, are responsible for the observed effect. The permanent damage or degradation was severe in case of fracture toughness and flexural modulus. Flexural strength of both systems was relatively unaffected by the absorption-desorption cycle. Permanent damage was found to be the highest for Somasif MAE clay reinforced specimens between two clay-epoxy nanocomposite systems. After studying the SEM micrographs of the fracture surfaces, it was speculated that moisture absorption had higher negative impact on the interface of Somasif MAE clay and epoxy matrix compared to the other clay-epoxy system. The hydrophobic nature of the Somasif MAE clay due to the presence of –F (fluorine) in the structure may have created additional tension between the polymer crosslinks in presence of moisture. FTIR spectra of the clay particles treated with the same absorption-desorption cycle provided proof that both nanoparticles undergo minimal chemical change and retain their respective original chemistry. This observation makes the aforementioned speculation more plausible. Although incorporation of clay in epoxy matrix did not fully stop the degradation, it had positive effects to some extent. It was observed that the studied properties in general were less severely degraded for clay-epoxy nanocomposites compared to neat epoxy samples. Therefore, clay particles could be successfully used to reinforce polymer materials to reduce the severity of property deterioration caused by the moisture absorption. However, the chemistry between the clay particles and polymer matrix and more specifically the chemical structure of the clay particles should be carefully considered to attain the best possible resistance against the property deterioration.

## Figures and Tables

**Table 1:** Structure of the studied clay particles.

Clay	Structure
Nanocor I.28E	(Na)_*y*_(Al_2−*y*_Mg_*y*_)(Si_4_O_10_)(OH)_2_.*n*H_2_O
	0.25 < *y* < 0.6

Somasif MAE	(Na)_2*x*_(Mg)_3−*x*_(Si_4_O_10_)(F_*a*_OH_1−*a*_)_2_.*n*H_2_O
	0.15 < *x* < 0.5
	0.8 < *a* < 1.0

**Table 2:** Weight changes in samples after preconditioning.

Specimens	Absorption, 24 hr (%)	Desorption, 6 hr (%)	Removal (%)
Neat EPON 862	2.10	0.14	93.35

0.5 wt% Nanomer I.28E	2.09	0.40	83.65
1.0 wt% Nanomer I.28E	2.15	0.42	80.33
1.5 wt% Nanomer I.28E	2.13	0.44	79.56
2.0 wt% Nanomer I.28E	2.20	0.45	79.35

0.5 wt% Somasif MAE	2.14	0.32	85.18
1.0 wt% Somasif MAE	2.12	0.35	83.70
1.5 wt% Somasif MAE	2.11	0.40	81.21
2.0 wt% Somasif MAE	2.13	0.45	79.07
